# Comparative impact of Roux-en-Y gastric bypass, sleeve gastrectomy or diet alone on beta-cell function in insulin-treated type 2 diabetes patients

**DOI:** 10.1038/s41598-024-59048-w

**Published:** 2024-04-08

**Authors:** Matthias Lannoo, Caroline Simoens, Roman Vangoitsenhoven, Pieter Gillard, André D’Hoore, Mieke De Vadder, Ann Mertens, Ellen Deleus, Nele Steenackers, Chantal Mathieu, Bart Van der Schueren

**Affiliations:** 1 Department of Abdominal Surgery, University Hospitals Leuven, Leuven, Belgium; 2 Clinical and Experimental Endocrinology, Department of Chronic Diseases and Metabolism, KU Leuven, Leuven, Belgium; 3 Laboratory of Ion Channel Research, Department of Cellular and Molecular Medicine, KU Leuven, Leuven, Belgium; 4 Department of Endocrinology, University Hospitals Leuven, Herestraat 49, 3000 Leuven, Belgium

**Keywords:** Obesity, Type 2 diabetes

## Abstract

Although bariatric surgery is an effective treatment for type 2 diabetes by inducing weight loss and augmenting gut hormone secretion, the immediate effect on beta-cell function itself remains to be elucidated in type 2 diabetes. Therefore, a prospective, randomized trial was performed in 30 patients with insulin-treated type 2 diabetes and a body mass index ≥ 35 kg/m^2^. Patients were randomly assigned (1:1:1) to Roux-en-Y gastric bypass (RYGB) or sleeve gastrectomy (SG) in combination with protein-sparing modified fast (PSMF), or to PSMF alone. Eu- and hyperglycemic clamps were performed before and 3 weeks after surgery and/or PSMF initiation. The primary outcome was the evolution of insulin sensitivity and beta-cell function after surgery, calculated using the composite measures of glucose disposal rate, insulin secretion rate, and disposition index (DI). Results revealed that markers of insulin sensitivity increased similarly in all arms (*p* = 0.43). A higher marker for maximal beta-cell function was observed when comparing SG to PSMF (*p* = 0.007). The DI showed a clear positive evolution after RYGB and SG, but not after PSMF alone. Altogether, these findings indicate that bariatric surgery results in an immediate beta-cell function recovery in insulin-treated type 2 diabetes.

## Introduction

Bariatric surgery exerts powerful effects on glucose homeostasis through weight loss alongside a plethora of metabolic and endocrine changes^1,2^. Although the underlying mechanisms are not fully understood, weight loss is often considered the main driver of improving insulin sensitivity. Nutritional approaches (i.e. a very low-calorie diet) leading to weight loss have been shown to facilitate sustained remission of type 2 diabetes, if subsequent weight regain is avoided^3–5^. Meanwhile, a relapse in diabetes has been linked to weight regain^6^. Interestingly, bariatric surgery exerts important weight-independent effects on glucose homeostasis through the enhanced post-prandial secretion of gut hormones [e.g., glucagon-like peptide 1 (GLP-1), peptide YY, and glucose-dependent insulinotropic polypeptide] before a significant amount of body weight is even lost^2,7–12^. These hormones affect beta-cell sensitivity and improve insulin secretion, which has led to the inclusion of bariatric surgery in the treatment paradigm of type 2 diabetes^13,14^.

In recent years, interest has grown in whether bariatric procedures themselves improve the hallmarks of the disease, impaired insulin sensitivity, and beta-cell function, directly^15^. Although some studies have investigated the effect of bariatric surgery, there remains controversy through heterogeneity in applied methodology (e.g., oral glucose tolerance tests, mixed meal tolerance tests, or glucose clamps), and postoperative timing^8,10,11,16,17^. Furthermore, previous studies have not characterized the metabolic repercussions across the full spectrum of patients with type 2 diabetes as studies often excluded individuals receiving insulin therapy^7,8,10,16^. In the case of non-insulin-dependent type 2 diabetes, beta-cell function is still relatively well preserved^8–10^. Hence, it remains uncertain whether bariatric surgery might restore the beta-cell function of patients with insulin-treated type 2 diabetes. Therefore, a randomized controlled trial was performed in patients with insulin-treated type 2 diabetes to measure beta-cell function and insulin sensitivity before and immediately after Roux-en-Y gastric bypass (RYGB) or sleeve gastrectomy (SG).

## Methods

### Study design and participants

A prospective, open-label, randomized study was performed at the Obesity Clinic of the University Hospitals of Leuven, Belgium. Patients were screened and considered eligible if they met all inclusion criteria (Table 1) including but not limited to having an age between 18 and 65 years, a body mass index (BMI) ≥ 35 kg/m^2^, being diagnosed with type 2 diabetes necessitating intensive insulin therapy using multiple daily injections. Insulin therapy was defined as requiring either basal or bolus insulin injections for effective diabetes management. Furthermore, eligibility required candidates to have been approved for bariatric surgery by the local multidisciplinary obesity team in accordance with the National Institutes of Health guidelines^18^. Informed consent was obtained from all individual participants included in the study. The study was approved by the local Ethics Committee Research UZ / KU Leuven, registered on ClinicalTrials.gov (NCT01086111), and conducted in accordance with the Declaration of Helsinki.

**Table 1:** Eligibility criteria.

*Inclusion criteria*
Female or male subjects aged 18–65 years BMI ≥ 35 kg/m^2^ Patient suffers from type 2 diabetes necessitating intensive insulin therapy Subjects are capable and willing to give informed consent Subjects are non-smokers for at least 6 months prior to the study start Use of oral, injected or implanted hormonal methods of contraception from at least the commencement of the last normal period prior to the screening visit in case of female patients of child bearing potential. Patients using hormonal contraception were advised to use a barrier method in addition from screening visit until their next normal period following the end of the study Indication for surgery is approved by the local multidisciplinary obesity workgroup following the NIH guidelines of 1991^18^
*Exclusion criteria*
Female patient is pregnant or breastfeeding BMI < 35 kg/m^2^ Patient suffers from an endocrine disease besides type 2 diabetes and thyroid disease, such as Cushing’s disease, Addison’s disease, hypothalamic tumor, … Patient suffers from type 1 diabetes, MODY or LADA Patient has undergone previous surgical procedure for weight loss Patient is considered ASA 4 or more according to the ASA physical status classification system of the American Society of Anesthesiologists Patient suffers from liver cirrhosis Patient uses steroids Patient uses cyclosporine Recent (< 30 days) or simultaneous participation in another clinical trial Any situation that can compromise the study, including serious illness or a predictable lack of cooperation from the subject

*ASA* American Society of Anesthesiology, *BMI* body mass index, *MODY* maturity-onset diabetes of the young, *NIH* National Institutes of Health, *LADA* latent auto-immune diabetes in adults.

### Randomization and blinding

Eligible patients were randomly assigned to a protein-sparing modified fast (PSMF) diet alone, or RYGB or SG in combination with PSMF diet in an allocation of 1:1:1 by a person unaffiliated with the clinical trial. Due to the nature of the intervention, blinding of treatment allocation was not possible. Once all study procedures were performed, all patients randomized to PSMF only also underwent bariatric surgery.

### Surgical and dietary intervention

The surgical interventions were performed in a single center by the same experienced surgeon using an already-established technique. Briefly, the laparoscopic RYGB procedure consisted of the formation of a small gastric pouch with a 25 mm circular stapled outlet, an alimentary limb of 120 cm, and a biliary limb of 30 cm. A laparoscopic SG procedure consisted of a resection of the fundus, corpus, and a part of the antrum of the stomach. This was calibrated with a 32 French gastric tube and stapled from a point, halfway between the incisura angularis and the pylorus, to the angle of Hiss. Regarding the dietary intervention, all participants were subjected to a PSMF diet (Modifast^®^ Intensive, Nutrition & Santé, Belgium) with a daily caloric content of 800 kcal (i.e. very-low calorie diet). All surgical patients received the PSMF diet from the first postoperative day until three weeks after surgery, while the non-surgical patients were subjected to the same PSMF diet for three consecutive weeks. At the time of the intervention, bolus long-acting insulin glargine (Lantus^®^, Sanofi, France) was replaced by NPH insulin (Insulatard^®^, Novo Nordisk, Denmark) to 50% of the normal dose in all participants irrespective of group assignment. This alteration was performed to mitigate the risk of hypoglycemia when the PSMF diet was initiated, either after surgery or following the baseline clamp in the PSMF group. In addition, basal insulin doses were halved in the surgical groups to account for the immediate postoperative dietary adjustments and metabolic effects of surgery, but maintained at the same dose in the PSMF group.

### Clamp procedures

**Figure 1:**
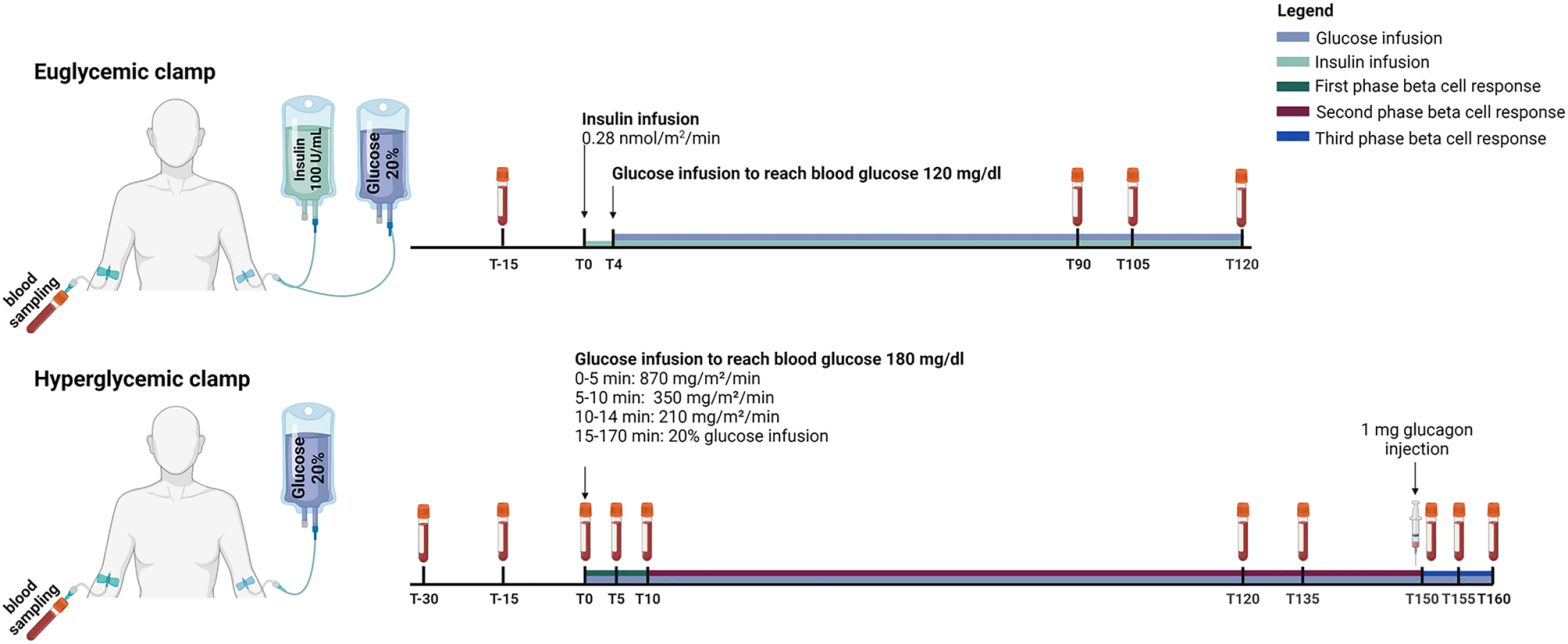
Protocol of the euglycemic and hyperglycemic clamp performed before and three weeks after each intervention. The euglycemic clamp (upper panel) involves a continuous insulin infusion (0.28 nmol/m^2^/min), adjusted via variable 20% glucose infusion to maintain a blood glucose level of 120 mg/dL based on 5-min interval bedside glucose measurements. Blood samples were taken at − 15, 90, 105, and 120 min to assess insulin and glucose. The hyperglycemic clamp (lower panel) raises blood glucose to 180 mg/dL using graded glucose infusions between T0 and T170 with blood samples at − 30, − 15, 0 to measure baseline glucose, insulin, and C-peptide before glucose infusion is started. Consequently, blood samples were collected at 5, 10, 120, 135, 150, 155, and 160 min to measure glucose, insulin, and C-peptide to evaluate first, second, and third phase beta-cell responses, respectively. Glucagon (1 mg) was injected at 150 min to assess maximal secretory capacity.

All patients were subjected to a euglycemic clamp (day − 4) followed by a hyperglycemic clamp (day − 1) before surgery or PSMF initiation (day 0), and three weeks after surgery or diet initiation (days 21 and 24). After an overnight fast of at least 12 h, the euglycemic and hyperglycemic clamps were performed according to a modified version of the protocol of DeFronzo et al*.*^19,20^. The overall approach of each clamp is outlined in Fig. 1. On the day of the clamp, intake of all medications was deferred until after the procedure.

To perform the euglycemic clamp (Fig. 1—upper panel), intravenous catheters were inserted into antecubital veins in both arms for the infusion of insulin (Actrapid^®^ 100 units/mL, Novo Nordisk, Denmark) and a 20% glucose solution, and for intermittent blood sampling. Blood sampling was performed before insulin infusion (− 15 min) for the determination of glucose and insulin. Afterward, insulin was infused with a constant infusion rate of 0.28 nmol/m^2^/min from 0 to 120 min. After four minutes, a 20% glucose infusion was added at a variable rate to maintain the blood glucose target concentration of 120 mg/dL. The rate of infusion was adjusted based on bedside blood glucose measurement occurring every 5 min (Hemocue^®^, Sweden). Blood samples were drawn during the steady state condition at 90, 105, and 120 min for measurement of glucose and insulin. Each patient was invited three days later to perform the hyperglycemic clamp (Fig. 1—lower panel). Similarly, intravenous catheters were inserted into the antecubital veins in both arms for the infusion of a 20% glucose solution and intermittent blood sampling. To measure the baseline beta-cell response (phase 0: − 30 to 0 min), blood samples were obtained at − 30, − 15, and 0 min for the determination of glucose, insulin, and C-peptide before glucose infusion. Afterward, plasma glucose was acutely raised to 180 mg/dL in approximately 14 min using a fixed dose of 870 mg/m^2^/min (time: 0–5 min), 350 mg/m^2^/min (time: 5–10 min), 210 mg/m^2^/min (time: 10–14 min) and 180 mg/m^2^/min (after 14 min). Bedside blood glucose measurement was performed every 5 min (Hemocue^®^, Sweden) to stabilize hyperglycemia during the first 30 min. From 15 to 150 min, the blood glucose target concentration of 180 mg/dL was maintained by infusing the 20% glucose solution at a variable rate based on the bedside blood glucose measurement occurring every 5 min. Afterward, the glucose infusion rate at 150 min was maintained until 170 min and an intravenous injection of 1 mg glucagon was given. During the clamp procedure, blood samples were collected at 5, 10, 120, 135, 150, 155, and 160 min for the determination of glucose, insulin, and C-peptide concentrations to evaluate first (phase 1: 0–10 min), second (phase 2: 10–150 min) and third phase beta-cell response or maximal secretory capacity (phase 3: 150–160 min).

### Laboratory assays

Plasma glucose was analyzed with a colorimetric hexokinase UV assay using COBAS 8000 (Roche^®^, Basel, Switzerland), while plasma insulin and C-peptide were measured by a chemiluminescent immunoassays on a COBAS 8000 Analyzer (Roche^®^, Basel, Switzerland).

### Primary outcomes

The primary outcome of the study was the evolution of beta-cell function and insulin sensitivity after RYGB, SG, and PSMF alone. Beta-cell function was measured using the disposition index (DI), while insulin sensitivity was measured using the glucose disposal rate (GDR).

### Data analysis and statistical analysis

From the hyperglycemic clamp, the AUC of C-peptide was expressed for each phase and expressed per minute. The insulin secretion rate (ISR) was calculated from plasma C-peptide levels using the Insulin SECretion (ISEC) method, of which ISR phase 3 is used as the index of maximal beta-cell function^21–23^. From the euglycemic clamp, the steady-state glucose infusion rate was used to evaluate whole-body insulin sensitivity expressed as GDR. The DI was then calculated as an integrated measure of beta-cell function using the phase 3 AUC of the ISR of the hyperglycemic clamp multiplied by the GDR of the euglycemic clamp.

Statistical analyses were performed using SAS software, version 9.2 of the SAS System for Windows. A convenience sample of 30 patients was included in this exploratory study, with 10 patients allocated to each of the three intervention groups. Data are presented as mean ± SD unless otherwise stated. Between-group comparisons of pre-intervention characteristics and post-intervention weight loss were performed using one-way ANOVA, followed by post-hoc Tukey tests for pairwise comparisons. Within-group comparisons of pre- and post-intervention characteristics were performed using paired t-tests. To compare post-intervention AUC values between groups, ANCOVA was performed with pre-intervention values as a covariate, and Tukey adjustments were used for pairwise comparisons between groups. Least square means with the corresponding 95% confidence intervals (CI) were reported. Statistical significance was set at *p* < 0.05 (two-tailed).

## Results

### Study participants

Between June 2010 and September 2014, a total of 34 patients were randomized to the trial (RYGB: *n* = 14; SG: *n* = 10 and PSMF:* n* = 10). Out of the patients assigned to RYGB, two withdrew early from further study participation and two had to be excluded due to complications of bariatric surgery.

Table 2 displays the patient’s characteristics per group before and after each intervention. Despite randomization, patients allocated to PSMF had a lower BMI, higher HbA_1_C, and a longer duration of type 2 diabetes when compared to both surgical groups (all: *p* < 0.05). Before every intervention, all patients suffered from insulin-treated type 2 diabetes with poor glycemic control (mean HbA_1c_ ± SD: 8.6 ± 1.7%). After every intervention, bolus insulin doses were less than half of the pre-intervention doses (all: *p* < 0.05; per protocol). A significant reduction was observed in basal insulin doses after each surgical intervention, (for SG and RYGB: *p* < 0.05; per protocol), but not in the PSMF group.

**Table 2:** Patient’s characteristics pre-intervention and 3 weeks post-intervention.

	Pre-intervention			3 Weeks post-intervention		
	PSMF (*n* = 10)	RYGB (*n* = 10)	SG (*n* = 10)	PSMF (*n* = 10)	RYGB (*n* = 10)	SG (*n* = 10)
Age (years)	60.6 ± 3.9	52.5 ± 7.9	52.9 ± 10.0	NA	NA	NA
Female (*n* (%))	8 (80)	5 (50)	4 (40)	NA	NA	NA
BMI (kg/m^2^)	38.7 ± 4.1*	41.3 ± 6.6	40.1 ± 7.9	37.0 ± 4.1^†^	37.0 ± 5.7^†^	36.8 ± 7.5^†^
Weight (kg)	105.2 ± 17.0	118.8 ± 18.2	122.4 ± 28.4	100.5 ± 15.8^†^	106.3 ± 15.2^†^	112.2 ± 25.9^†^
Weight loss (%)	NA	NA	NA	4.4 ± 1.8^‡^	10.4 ± 2.7	8.4 ± 2.7
Diabetes duration (years)	16.3 ± 8.3*	9.3 ± 7.8	13.5 ± 8.4	NA	NA	NA
HbA_1c_ (%)	9.3 ± 1.6*	8.0 ± 1.8	8.7 ± 1.6	NA	NA	NA
HbA_1c_ (mmol/mol)	78 ± 18	64 ± 20	72 ± 18	NA	NA	NA
Metformin (*n* (%))	9 (90)	8 (80)	10 (100)	7 (70)	7 (70)	10 (100)
Sulfonylurea (*n* (%))	0 (0)	0 (0)	0 (0)	0 (0)	0 (0)	0 (0)
GLP-1 agonist (*n* (%))	0 (0)	1 (10)	0 (0)	0 (0)	0 (0)	0 (0)
DPP-IV inhibitor (*n* (%))	0 (0)	0 (0)	0 (0)	0 (0)	0 (0)	0 (0)
Insulin bolus (IU)	49.4 ± 41.3	37.1 ± 19.1	52.9 ± 23.5	22.4 ± 18.4^†^	14.2 ± 7.7^†^	17.8 ± 15.8^†^
Insulin basal (IU)	48.0 ± 19.9	42.1 ± 13.5	40.4 ± 19.1	45.5 ± 21.2	17.0 ± 4.1^†^	29.1 ± 14.1^†^
Fasting glucose (mg/dL)	115.2 ± 10.0	109.0 ± 9.3	116.1 ± 15.5	107.5 ± 14.9	106.3 ± 17.6	95.2 ± 11.5^†^
Fasting C-peptide (pmol/L)	407.7 ± 145.7	648.2 ± 427.5	691.9 ± 347.0	501.7 ± 175.4	780.2 ± 333.6	760.6 ± 233.5
Fasting insulin (pmol/L)	317.6 ± 343.4	179.4 ± 174.3	217.6 ± 166.1	234.9 ± 179.1	117.3 ± 59.1	78.4 ± 43.0^†^

Pre-intervention values were compared using ANOVA (*p < 0.05) with Tukey adjustments for pairwise comparisons. Post-intervention values were compared to pre-intervention using a paired Student’s t-test (^†^*p* < 0.05). Weight loss at 3 weeks post-intervention was compared using ANOVA (^‡^*p* < 0.05) with Tukey adjustments for pairwise comparisons (*p* < 0.001 RYGB vs. PSMF, *p* = 0.001 SG vs. PSMF, *p* = 0.13 RYGB vs. SG). Data are mean ± SD, unless otherwise stated. *DPP-IV* dipeptidylpeptidase IV, *GLP-1* glucagon-like peptide 1, *NA* not applicable, *PSMF* protein-sparing modified fast, *RYGB* Roux-en-Y gastric bypass, *SG* sleeve gastrectomy.

### Insulin production, secretion and sensitivity

**Figure 2:**
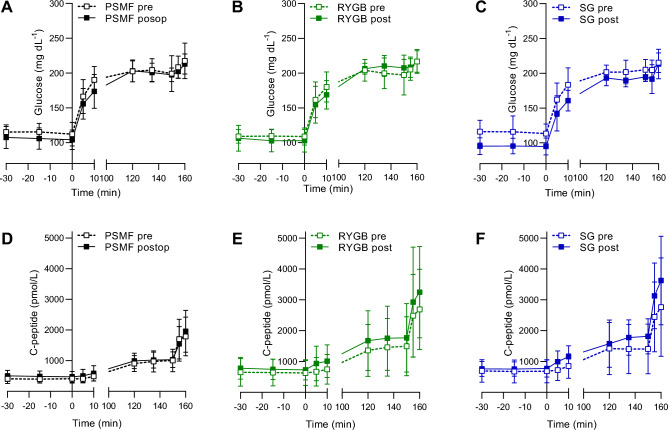
Evolution of glucose and C-peptide during the hyperglycaemic clamp pre- and post-intervention. Glucose during the hyperglycaemic clamps pre-intervention and 3 weeks post-intervention with (**a**) PSMF (*n* = 10), (**b**) RYGB (*n* = 10) and (**c**) SG (*n* = 10). C-peptide during the hyperglycaemic clamps pre-intervention and 3 weeks post-intervention with (**d**) PSMF, (**e**) RYGB and (**f**) SG. Data are presented in mean ± SD. PSMF: protein-sparing modified fast; RYGB: Roux-en-Y gastric bypass; SG: sleeve gastrectomy.

Before the intervention, the AUC of C-peptide levels was not statistically different between groups at baseline (phase 0), after acute glucose stimulation (phase 1), in the steady state (phase 2), or after maximal stimulation with glucagon (phase 3) of the hyperglycemic clamp procedure (Supplementary Table S1). Regarding insulin secretion, pre-intervention ISR was not statistically different between the three groups (Supplementary Table S1). Regarding insulin sensitivity, no differences in GDR were observed between the groups during the post-intervention euglycemic clamp (*p* = 0.63; Supplementary Table S1). The pre- and post-intervention evolution of glucose and C-peptide levels during the hyperglycaemic clamp are visualized in Fig. 2, which was characterized by a steep rise after glucose bolus, followed by a more stable plateau and a final steep rise after glucagon injection.

Post-intervention values for C-peptide and ISR in all phases of the hyperglycemic clamp and GDR values of the euglycemic clamp are summarized in Table 3. Regarding insulin production, the AUC of C-peptide was not statistically different between groups at baseline (phase 0), after acute glucose stimulation (phase 1), or in the steady state (phase 2) of the hyperglycemic clamp procedure at 3 weeks post-intervention, (Fig. 2; Table 3). Nor were C-peptide levels different at any time point after intervention (Fig. 2). However, the AUC for C-peptide differed between the three groups after maximal stimulation with glucagon during phase 3 (PSMF diet: 1886 (1445; 2327) pmol/L/min; RYGB: 2578 (2150; 3007) pmol/L/min; SG: 2607 (2174; 3039) pmol/L/min (*p* = 0.045). Pairwise comparisons showed a trend for a higher AUC C-peptide when comparing both surgery groups to the PSMF diet alone (*p* = 0.08 and 0.07 for RYGB and SG vs. PSMF alone, respectively). Regarding insulin secretion, post-intervention ISR differed significantly between the three groups during phases 2 and 3 of the hyperglycemic clamp (PSMF diet: 3 (3; 4) and 9 (6; 12) pmol/kg/min; RYGB: 5 (4; 6) and 12 (9; 15) pmol/kg/min; SG: 5 (5; 6) and 15 (13; 18) pmol/kg/min; *p* = 0.009 and 0.010 for phase 2 and 3, respectively). Pairwise comparison showed the presence of a higher ISR in both phases 2 and 3 of the hyperglycemic clamp following SG compared to the PSMF diet alone (*p* = 0.008; *p* = 0.007; respectively). Moreover, a trend for a higher ISR was observed in phase 2 of the hyperglycemic clamp following RYG compared to PSMF diet alone (*p* = 0.06). Regarding insulin sensitivity, no differences in GDR were observed between the groups during the post-intervention euglycemic clamp (*p* = 0.43) (Table 3).

**Table 3:** The post-intervention AUC of C-peptide, ISR and GDR.

		Least square means (95% CI)			ANCOVA	Pairwise comparisons (Tukey)		
		PSMF (*n* = 10)	RYGB (*n* = 10)	SG (*n* = 10)		RYGB versus PSMF	SG versus PSMF	RYGB versus SG
AUC C-peptide (pmol/L/min)	Phase 0	588 (467;710)	712 (596;829)	707 (589;825)	0.28	0.31	0.36	1.00
	Phase 1	637 (437;836)	881 (692;1070)	880 (684;1076)	0.16	0.19	0.22	1.00
	Phase 2	1267 (1028;1506)	1600 (1367;1834)	1626 (1393;1859)	0.07	0.13	0.09	0.99
	Phase 3	1886 (1445;2327)	2578 (2150;3007)	2607 (2174;3039)	**0.045**	0.08	0.07	0.99
ISR (pmol/kg/min)	Phase 0	2 (1;2)	2 (2;2)	2 (2;2)	0.26	0.30	0.34	1.00
	Phase 1	2 (1;3)	3 (2;4)	4 (3;5)	0.10	0.31	0.08	0.71
	Phase 2	3 (3;4)	5 (4;6)	5 (5;6)	**0.009**	0.06	**0.008**	0.70
	Phase 3	9 (6;12)	12 (9;15)	15 (13;18)	**0.010**	0.17	**0.007**	0.28
GDR (mg/kg/min)		1.70 (0.89;2.52)	1.69 (0.87;2.50)	2.34 (1.52;3.15)	0.43	1.00	0.50	0.49

Post-intervention values for C-peptide and ISR in all phases of the hyperglycemic clamp and GDR values of the euglycemic clamp. Comparison of the post-intervention values between all groups was performed by ANCOVA with the pre-intervention value as covariate. Tukey adjustments were used for pairwise comparisons between groups. *GDR* glucose disposal rate, *ISR* insulin secretion rate, *PSMF* protein-sparing modified fast; RYGB Roux-en-Y gastric bypass, *SG* sleeve gastrectomy.

Significant values are in bold.

### Disposition index

**Figure 3:**
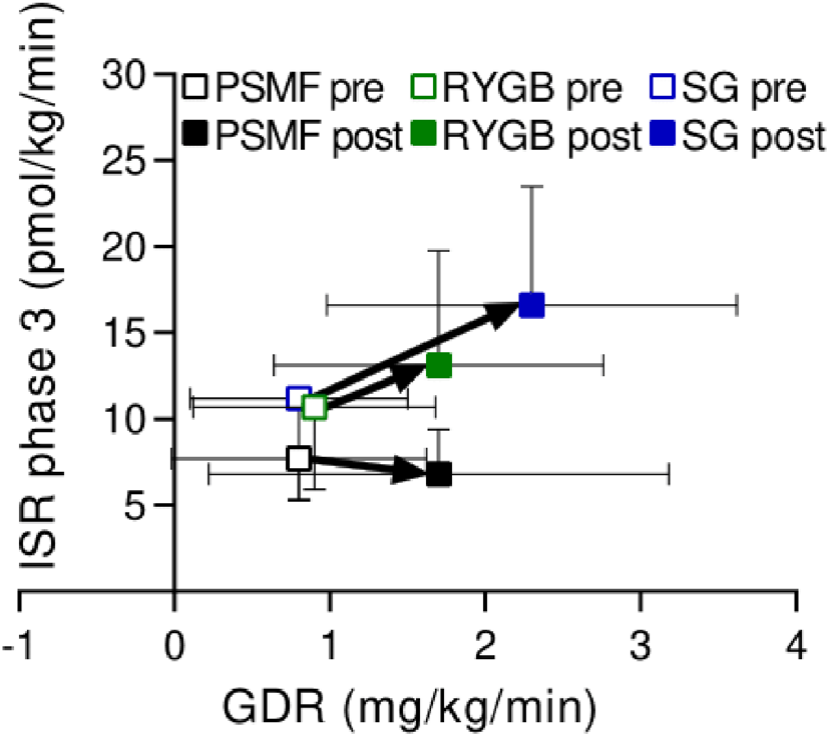
Evolution of the disposition index from pre- to post-intervention for PSMF (*n* = 10), RYGB (*n* = 10) and SG (*n* = 10). Insulin secretion rate during phase 3 of the hyperglycemic clamps vs glucose disposal rate (GDR) of the euglycemic clamps. Data are presented as mean ± SD. GDR: glucose disposal rate; ISR: insulin secretion rate; PSMF: protein-sparing modified fast; RYGB: Roux-en-Y gastric bypass; SG: sleeve gastrectomy.

Regarding beta-cell function, Fig. 3 visualizes the DI for phase 3 of the hyperglycemic clamp expressed as the ISR phase 3 versus GDR for both pre- and post-intervention. A clear positive evolution (right and upward) is noted for both surgical intervention groups, suggesting improved insulin sensitivity and heightened insulin secretion capacity. In contrast, the PSMF group only presented a rightwards shift without an increase in phase 3, reflecting improved insulin sensitivity without clear improvement in beta-cell function.

## Discussion

This randomized controlled trial evaluated the immediate effect of RYGB, SG, or PSMF diet alone on beta-cell function in 30 patients with obesity and insulin-treated type 2 diabetes using a euglycemic clamp and a hyperglycemic clamp first in absence and then in the presence of glucagon. Beta-cell function was addressed as a composite measure using a reflection of both insulin secretion (i.e. phase 3 AUC of the ISR) and sensitivity (i.e. GDR). Insulin secretion improved at three weeks after each surgical intervention, while no improvement was observed after a mere caloric restriction. Insulin sensitivity improved to the same extent after each intervention.

These findings provide important evidence to the current understanding of the improved beta-cell function after bariatric surgery^24^. Indeed, several studies with heterogenous enrollment criteria and methodology, and a recent systematic review (excluding hyperglycemic clamps) are available^8,10,25–29^. However, our findings are unique in terms of the study population, postoperative timing, and methodology. Regarding the study population, beta-cell function was investigated in patients with insulin-dependent type 2 diabetes and poor glycemic control. Until now, previous research focused on patients without type 2 diabetes, or patients with type 2 diabetes with relatively well-preserved beta-cell function (insulin-independent) that are in the early stages of the disease process^8,10,27,29–31^. In some cases, information regarding the extent of beta-cell function or diabetes duration was missing^25,26,32^. In case of good beta-cell function or shorter diabetes duration, there is a higher chance of achieving diabetes remission. However, the effect on the beta-cell function itself is bound to be limited as the room to improve is less than in patients with long-standing diabetes and poor beta-cell functional reserve. For this reason, it is often the ideal condition to be studied with the highest chance of demonstrating an effect^33^. Unfortunately, these inclusion criteria limit the applicability of the result towards the full spectrum of patients with type 2 diabetes. Regarding postoperative timing, the immediate effect of bariatric surgery was investigated to minimize the impact of differential weight loss and food intake between groups. Although weight loss was more pronounced after the surgical interventions, a similar improvement in insulin sensitivity was observed as demonstrated by the comparable increase in GDR after each intervention. These results enabled us to assess the isolated effect of both bariatric procedures on beta-cell responsiveness to hyperglycemia, eliminating major confounders like beta-cell stress differences due to a distinct insulin sensitivity. Regarding methodology, the majority of available studies applied oral stimuli during oral glucose tolerance tests (OGTT) or mixed meal tolerance tests (MMT). These techniques primarily engage the enteroinsulinar axis that starts with food ingestion^10,11^. After surgery, nutrient handling is profoundly altered due to procedure-specific anatomical rearrangement of the gastrointestinal tract with important physiological implications^34^. Altogether, nutrients will arrive more rapidly in the small intestine for absorption. This will lead to a short, high burst of serum glucose that triggers an acute release of ready-made insulin from secretory granules^35,36^. Thereby, these techniques using solely oral stimuli provide a picture of acute beta-cell secretory capacity rather than complete beta-cell function^37^. In our study, the gold standard hyperglycemic clamp was applied to evaluate beta-cell function^24^. By doing so, the well-characterized changes in glucose fluctuations and incretin hormones are circumvented that occur in response to oral stimuli elicited by an OGTT or MMTT after RYGB and SG^9,10,36,38,39^. Using this approach, a maximal secretory response from the beta-cells is provoked by incorporating an intravenous glucagon stimulation at the end of the clamp. This readout reflects the beta-cells' maximal responsiveness to hyperglycemia, which is less influenced by the surgical changes in gastrointestinal anatomy, and more reflective of the intrinsic pancreatic function. Using a hyperglycemic clamp, Kashyap et al.^17^ observed a modest increase in C-peptide secretion during phases 1 and 2 in people at four weeks after adjustable gastric banding and SG compared to RYGB. Our findings indicate an improvement in later stages of the hyperglycemic clamp (i.e. phase 2 and 3 for ISR, and phase 3 for AUC C-peptide), which could be explained by differences in the spectrum of patients with type 2 diabetes studied. The previous study investigated patients with type 2 diabetes, who were mainly glucose-tolerant with a shorter diabetes duration rather than insulin-dependent. When the beta-cell function is severely impaired, it is possible that restoration of the beta-cell response to hyperglycemia first occurs in the later stages of the clamp when the hyperglycemic stimulus has maximized, although this hypothesis requires further testing. Altogether, our findings indicate that bariatric surgery directly improves beta-cell function in patients with insulin-treated type 2 diabetes. This is particularly interesting as current treatment options mainly focus on improving insulin sensitivity rather than restoring beta-cell function to improve glycemic control.

A limitation of our exploratory study was the small sample size of patients, followed by the differences in baseline characteristics despite the fact that patients were randomized for intervention. It is possible that the longer duration of type 2 diabetes, poorer glycemic control at baseline, and lower weight loss in the PSMF group compared to the surgical groups might downplay the effect of caloric restriction alone on beta-cell function. Nonetheless, these pre-intervention differences were taken into account during statistical analysis. In light of the treatment paradigm of type 2 diabetes, there is a need to compare the postoperative endocrine and gastrointestinal function as different bariatric procedures present different stimuli to the pancreas and gut. From our study, it is challenging to compare outcomes between RYGB and SG due to the small study population. Nonetheless, two meta-analyses showed that both RYGB and SG are effective in improving diabetes outcomes with RYGB being potentially more superior for short-term diabetes remission and weight loss outcomes^33,40,41^. However, when the effects of RYGB and SG on beta-cell function were compared using dynamic metabolic perturbation in a meta-analysis comprising 7 studies, no clear superiority was observed for any procedure^28^.

Taken together, our exploratory results demonstrate that both RYGB and SG have the potential to partially restore beta-cell function in patients with insulin-treated type 2 diabetes and considerably impaired beta-cell function three weeks after surgery. These results are both promising and unique as improved beta-cell function after surgery is mainly attributed to an improved incretin effect, which was circumvented in our study through the use of an intravenous stimulus. It is worthwhile to investigate whether this short-term effect remains or even enhances years after surgery in a larger more balanced sample size that addresses the limitations of our exploratory study. Moreover, it is of prime interest to compare beta-cell function between responders and non-responders of bariatric surgery to identify whether this improvement is driving the force of surgery efficacy for diabetes remission.

While promising studies are potentially ongoing and needed, our findings further support a long-lasting place for bariatric surgery in the treatment paradigm of type 2 diabetes in case of insulin dependency.

### Supplementary Information

Supplementary Information.

## Data Availability

The datasets generated during and/or analyzed during the current study are available from the corresponding author on reasonable request.
