# Supplementary material for: Comparative impact of Roux-en-Y gastric bypass, sleeve gastrectomy or diet alone on beta-cell function in insulin-treated type 2 diabetes patients
Source: Sci Rep. 2024 Apr 8;14:8211. doi: 10.1038/s41598-024-59048-w (PMC11001928; doi:10.1038/s41598-024-59048-w)
Supplement: Supplementary file 1 — Supplementary Information. [file 41598_2024_59048_MOESM1_ESM.docx]

| **Table S1.** The pre-intervention AUC of C-peptide, ISR and GDR. | | | | | | | | |
| --- | --- | --- | --- | --- | --- | --- | --- | --- |
|  | | **Mean ± SD** | | | **P-Value** | **Pairwise comparisons (Tukey)** | | |
|  |  | **PSMF**  **(*n*=10)** | **RYGB**  **(*n*=10)** | **SG**  **(*n*=10)** |  | **RYGB**  **vs**  **PSMF**  **1-2** | **SG**  **vs**  **PSMF**  **1-3** | **RYGB**  **vs**  **SG**  **2-3** |
| **AUC C-peptide (pmol/L/min)** | **phase 0** | 403.4 ± 138.52 | 639.0 ± 428.00 | 683.1 ± 370.46 | 0.24 | 0.52 | 0.045 | 0.85 |
|  | **phase 1** | 429.8 ± 139.8 | 676.2 ± 450.9 | 810.4 ± 425.3 | 0.16 | 0.47 | 0.038 | 0.52 |
|  | **phase 2** | 966.0 ± 325.8 | 1441.7 ± 916.8 | 1408.7 ± 810.1 | 0.42 | 0.31 | 0.24 | 0.97 |
|  | **phase 3** | 1550.0 ± 553.7 | 2158.9 ± 1203.0 | 2265.0 ± 1141.5 | 0.38 | 0.52 | 0.14 | 0.73 |
| **ISR (pmol/kg/min)** | **phase 0** | 1.1 ± 0.2 | 1.5 ± 1.0 | 1.5 ± 0.9 | 0.39 | 0.63 | 0.09 | 1.00 |
|  | **phase 1** | 1.3 ± 0.3 | 2.0 ± 1.5 | 2.0 ± 1.0 | 0.29 | 0.62 | 0.07 | 0.67 |
|  | **phase 2** | 2.9 ± 0.8 | 4.3 ± 2.8 | 3.7 ± 2.06 | 0.61 | 0.43 | 0.41 | 0.85 |
|  | **phase 3** | 7.7 ± 2.4 | 10.7 ± 4.8 | 11.2 ± 5.9 | 0.15 | 0.11 | 0.09 | 0.94 |
| **GDR (mg/kg/min)** |  | 0.80 ± 0.82 | 0.90 ± 0.78 | 0.80 ± 0.70 | 0.63 | 0.39 | 0.47 | 1.00 |
| **Legend**: Pre-intervention values for C-peptide and ISR in all phases of the hyperglycemic clamp and GDR values of the euglycemic clamp. Comparison of the pre-intervention values between all groups was performed. Tukey adjustments were used for pairwise comparisons between groups. GDR: glucose disposal rate; ISR: insulin secretion rate; PSMF: protein-sparing modified fast; RYGB: Roux-en-Y gastric bypass; SG: sleeve gastrectomy. | | | | | | | | |
